# The polar code for patterning: how polarity and the cytoskeleton orchestrate asymmetric cell division during plant development

**DOI:** 10.3389/fcell.2025.1618444

**Published:** 2025-05-30

**Authors:** Akanksha Garhewal, Gabriel J. Angres, Andrew Muroyama

**Affiliations:** Department of Cell and Developmental Biology, Division of Biological Sciences, UC San Diego, La Jolla, CA, United States

**Keywords:** polarity, cytoskeleton, microtubule, F-actin, cell division, plant development

## Abstract

Cell polarity is fundamental to morphogenesis across living organisms. In plants, a dynamic interplay between polarity cues and the cytoskeleton orchestrates essential asymmetric cell divisions across diverse species. Here, we focus on three functions for the cytoskeleton—organelle positioning, cell growth and mitosis—and discuss our current understanding of how polarity controls these processes. By taking a comparative approach that highlights what is known about these pathways across plant species, we spotlight both the broadly conserved and cell type-specific ways that polarity can regulate division orientation. Because there have been significant developments in the field within the last several years, we focus our attention on recent work and give our perspective on exciting future avenues of investigation into the reciprocal relationship between polarity and the cytoskeleton.

## Introduction

Polarity imposes spatial information on a cell that is used to activate signaling cascades, organize the cytosol, and direct cell behavior. Polarization is itself a broad term that can refer to anisotropy at different levels. In this review, we focus our attention on polarized behaviors in asymmetrically dividing cells, which are essential for the development of many plant tissues (De Smet and Beeckman, 2011). In these cells, polarization is necessary to create two daughter cells of different identities and, often, sizes. While multiple definitions of polarity exist, polarization often refers to the creation of asymmetrically distributed, plasma membrane-associated protein complexes. These polarity domains scaffold the recruitment of effectors to control cellular dynamics, often in a cell cycle-dependent and tissue-specific manner. The list of proteins that are capable of polarizing continues to grow, revealing that some are relatively recent evolutionary innovations while others are conserved down to bryophytes (van Dop et al., 2020; Nir et al., 2022). The current challenge is now to define the mechanisms that link polar domain formation to downstream effects in the cell.

Mutant phenotypes upon polarity disruption are varied, but one that is shared in many loss-of-function and over-expression mutants is aberrant tissue patterning and associated cell division defects. Therefore, many groups have focused on understanding how polar domains control asymmetric cell divisions (ACDs). This regulation is mediated, in part, through polarization-enhanced signaling cascades, which promote division via effects on transcription and hormone responses (Zhang et al., 2015; Houbaert et al., 2018; Vukašinović et al., 2025). Here, however, we focus on a different facet of this regulation: polarity-mediated effects on the cytoskeleton that control division within developing tissues. As we highlight commonalities and important differences between pathways in different cell types, we point out future areas for investigation that we believe will be especially exciting in the coming years.

## Pre-division organelle positioning informs ACD orientation

**FIGURE 1:**
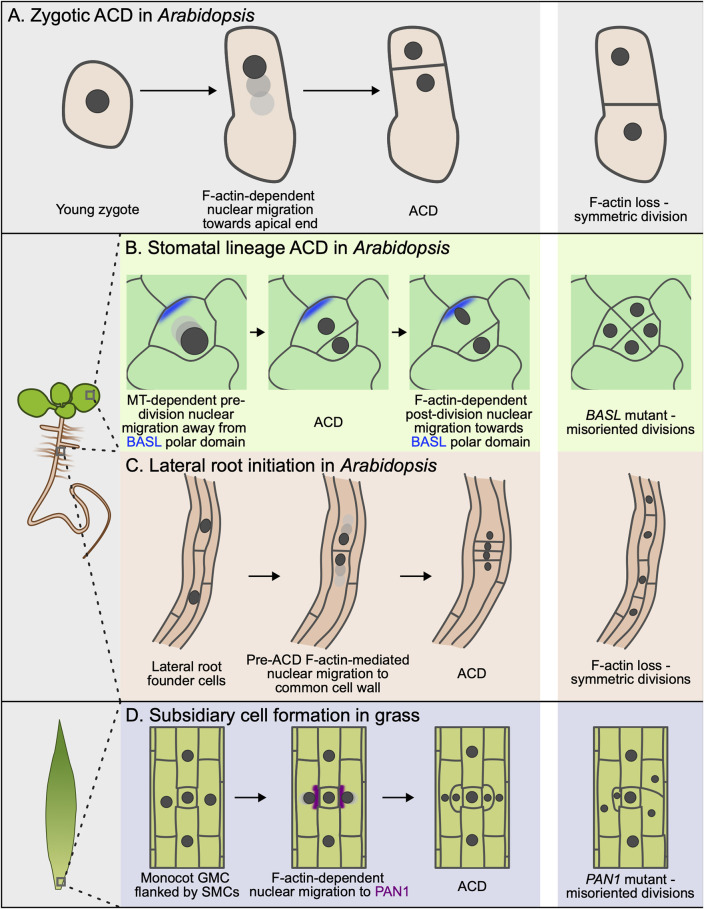
Nuclear migration and cell polarity during asymmetric cell division (ACD). **(A)** Following fertilization, the zygotic nucleus (dark grey) migrates toward the apical side of the cell. The zygote then undergoes an asymmetric division, producing a smaller apical cell and a larger basal cell. Disruption of F-actin using latrunculin B impairs nuclear migration, resulting in symmetric division. **(B)** Asymmetrically dividing cells in the *Arabidopsis* stomatal lineage use a cortical polarity domain defined by BASL (blue) to control two phases of nuclear migration. Before ACD, the nucleus migrates away from this domain in a microtubule-dependent manner. After division, the nucleus migrates toward the cortical polarity domain in an F-actin–dependent manner. Without *BASL*, directional nuclear migration is disrupted, leading to abnormal division patterns. **(C)** After specification, F-actin-dependent nuclear migration in lateral root founder cells is directed towards the shared cell wall. F-actin disruption results in more symmetric divisions and defective lateral root primordia. **(D)** A cortical polar domain classically defined by PAN1 (purple) forms at the interface between the guard mother cell (GMC) and SMC during subsidiary cell formation. The nucleus migrates toward this site in an F-actin-dependent manner. Disrupting polar domain formation in these cells leads to misoriented divisions and defective subsidiary cell formation.

Division orientation depends, in part, on the position of organelles before mitotic onset. More specifically, nuclear position instructs cell division by 1) scaffolding the site of the preprophase band (PPB) and 2) nucleating the site of spindle assembly upon nuclear envelope breakdown (Rasmussen and Bellinger, 2018). As such, most of the polarity-mediated ACDs studied to date are preceded by directional nuclear migration before PPB formation. While these migrations depend on cell cycle-regulated cytoskeletal reorganization, they can be directed either toward or away from the polar site, revealing that there is no universal mode of pre-ACD nuclear migration in plants (Figure 1). Instead, polarity can differentially remodel F-actin and/or microtubules to promote nuclear migration depending on the cell type and plant species.

### Stomatal development in grasses depends on pre-division nuclear migration

Stomatal formation in maize *(Z. mays*) has long been an excellent model for interrogating polarity-controlled nuclear migrations, and over two decades of research on this system have made it a valuable point of reference for other nuclear migrations. Stomatal complexes in grasses, such as *Zea mays*, are composed of four cells: paired guard cells (GCs) that generate the stomatal pore and a pair of flanking subsidiary cells (SCs) that facilitate pore opening and closing (Raissig et al., 2017; Pichaco et al., 2024). Two ACDs are required to generate these complexes. The first is a poorly understood ACD that generates the guard mother cell (GMC). Polar proteins that control this division have not been identified, but time-lapse imaging of a maize line harboring nuclear and microtubule reporters has shown that directed nuclear migration in the tip-ward direction precedes ACD, indicating that yet-unknown upstream cues control nuclear position (Ashraf et al., 2023). Future work will be required to clarify the underlying cytoskeletal regulators involved in this process.

Much more is known about the subsequent ACD that generates SCs from the subsidiary mother cells (SMCs) that flank the GMC. SC recruitment requires a pre-division nuclear migration in SMCs towards a polar domain that forms at the GMC/SMC contact site (Gallagher and Smith, 2000). The first step of this process is polar localization of BRICK1 (BRK1), a component of the SCAR/WAVE complex that activates the branched actin nucleating Arp2/3 complex (Frank and Smith, 2002; Frank et al., 2004; Facette et al., 2015). Along with the closely related BRK3, BRK1 recruits the leucine-rich repeat receptor-like kinases (LRR-RLKs) PANGLOSS1 (PAN1) and PANGLOSS2 (PAN2) (Cartwright et al., 2009; Zhang et al., 2012; Facette et al., 2015). PAN2, in turn, is required for polar localization of WEB1/PMI2-RELATED (WPR) proteins, which were shown to bind to F-actin (Nan et al., 2023a). Finally, subsequent recruitment of RHO GTPASES OF PLANTS 2 (ROP2) and ROP9 activates Arp2/3-dependent formation of an F-actin patch at the polarized contact site (Humphries et al., 2011) (Figure 1D). Mutants in any of these components result in SMC division orientation defects of varying severity, reinforcing that hierarchical recruitment of polarized proteins is necessary for stomatal development in maize. A recent study showed that loss of the *Brachypodium distachyon* PAN1 homolog, BdPAN1, similarly results in failed nuclear migration and ACD orientation defects, highlighting conservation of key regulators in these processes across different grass species (Zhang et al., 2022).

How does the polar domain control pre-division nuclear migration? An attractive hypothesis is that polarization promotes F-actin patch formation, which subsequently directs the nucleus to move toward the SMC/GMC contact site. Indeed, treatment with the F-actin depolymerizing drug latrunculin B eliminates F-actin patch formation and impairs nuclear migration (Panteris et al., 2006). Additionally, pre-division nuclear movement and subsidiary cell formation require Maize LINC KASH AtSINE-like2 (MLKS2), a KASH domain protein that links F-actin to the nuclear envelope (Gumber et al., 2019; Ashraf et al., 2023). Finally, F-actin is required for stochastic nuclear movements and other, directed nuclear migrations not associated with cell division, for example, during fertilization and in growing root hairs and pollen tubes (Kawashima et al., 2014; Brueggeman et al., 2022; Wang et al., 2024). However, nuclei were polarly localized in a significant fraction of SMCs that did not form an F-actin patch in *pan* mutants, suggesting that these two processes can be uncoupled (Cartwright et al., 2009). Therefore, even though polarization triggers F-actin reorganization and F-actin is required for nuclear migration, the precise connection between polarity-mediated F-actin reorganization and nuclear migration remains mysterious.

### Paired nuclear migrations control ACD orientation in the *Arabidopsis* stomatal lineage

Polarization is also a hallmark of the ACDs that create stomata in eudicots like *Arabidopsis*. Before each ACD, two opposing polar domains form at the plasma membrane. The more well-defined of the two contains BREAKING OF ASYMMETRY IN THE STOMATAL LINEAGE (BASL), BREVIS-RADIX family (BRXf) proteins, and POLAR LOCALIZATION DURING ASYMMETRIC DIVISION AND REDISTRIBUTION (POLAR) (Dong et al., 2009; Pillitteri et al., 2011; Rowe et al., 2019), which scaffold the recruitment of effectors including BSL1, BIN2, PRAF and YDA (Zhang et al., 2015; Houbaert et al., 2018; Guo et al., 2021; Wang et al., 2022). Collectively, the proteins in this BASL-containing domain ensure differential daughter cell fates. A second polar domain defined by the OCTOPUS-LIKE (OPL) proteins forms opposite the BASL domain and influences division potential (Wallner et al., 2023).

In addition to shaping cell identity, it has long been hypothesized that the BASL-containing polar domain might also control division orientation. This assumption had its roots in the observation that the smaller meristemoid is always generated distal to the site of BASL polarization, which suggested that BASL might orient the ACD. Recently, Muroyama et al. showed that each ACD is bookended by opposing nuclear migrations that are directed by the BASL domain (Muroyama et al., 2020) (Figure 1B). Interestingly, there are several key differences between these nuclear migrations in the *Arabidopsis* stomatal lineage and in SMCs in grasses. First, these two pre-division nuclear migrations are oppositely oriented with respect to the polar site; the BASL crescent repels the nucleus in *Arabidopsis* while the nucleus is attracted to the PAN1 domain in SMCs. Second, the pre-division nuclear migration in *Arabidopsis* depends on microtubules, not F-actin; treatment with the microtubule depolymerizing drug oryzalin blocks pre-division nuclear migration while latrunculin B treatment has no effect. Third, there is a second, post-ACD nuclear migration in *Arabidopsis* where the nucleus exhibits a striking, F-actin-dependent migration towards the BASL domain in the stomatal lineage ground cell (SLGC). Based on these observations, the post-division nuclear migration more closely resembles the pre-division nuclear migration in grass SMCs, while the pre-division nuclear migration in *Arabidopsis* appears to use a wholly separate mechanism. Recent work has shown that polarized BASL locally disrupts the cortical microtubule array (explained in more detail below) (Muroyama et al., 2023), although a direct connection between this effect on microtubule stability and pre-division nuclear migration has not been established. To date, it remains unclear if and how BASL influences F-actin organization to direct the post-division nuclear migration; fluorescent reporters of F-actin are not obviously enriched at the polar site and no F-actin regulators have been reported to polarize along with BASL. Careful characterization of changes to the F-actin and microtubule arrays before and after ACD will be important to clarify this process in the future.

### Nuclear migration precedes branching in moss

Microtubule-dependent pre-division nuclear migration has been described during protonemal branching in the moss *Physcomitrium patens*. Like the other nuclear migrations discussed thus far, the first step of this process is the formation of a polarized domain at the plasma membrane. In this case, a ROP polar domain forms a bulge within the subapical cell that attracts the nucleus (Yi and Goshima, 2020). Nuclear movement and branching are impaired in the higher order *rop2rop3rop4* mutant, and treatment with pharmacological inhibitors of F-actin and microtubules revealed that microtubules and F-actin are both required for this movement through different mechanisms. Microtubule depolymerization severely impacted nuclear movement without impacting formation of the ROP bulge. Conversely, latrunculin A treatment impeded bulging at the ROP domain and nuclear migration, although nuclear migration proceeded normally in plants that were treated with latrunculin A after bulge formation, indicating that bulge formation is upstream of nuclear migration but that F-actin is dispensable for the migration itself (Yi and Goshima, 2020).

Befitting its role in nuclear migration, the ROP domain is a site of cytoskeletal reorganization. F-actin fluorescent reporters are concentrated in the nascent bulge, linking polarization with local F-actin reorganization (Vidali et al., 2009). Tracking microtubule polymerization with a fluorescent EB1 reporter revealed that the majority of microtubule plus ends in this region grow into the bulge (Yi and Goshima, 2020). As a similar effect is seen in tip-growing cells in moss, the authors speculate that biased microtubule growth into the bulge could be a secondary consequence of cell geometry rather than a directed polymerization to that site (Yamada and Goshima, 2018). Taken together, these results highlight the potentially critical role cell morphology plays in reinforcing cytoskeletal organization to control nuclear migration.

### ACD-associated nuclear migrations in other polarized cell types

We have discussed examples of cells where polar domains are essential landmarks that direct nuclear migration. However, it is worth noting that nuclear migrations are not a universal feature of ACD. For example, nuclei are centrally positioned and do not migrate before undergoing ACD in the 8-cell *Arabidopsis* embryo (Vaddepalli et al., 2021). Conversely, there are critical ACDs that generate daughter cells with striking size asymmetry without a known associated polar domain. For example, new lateral root primordia are created by ACDs in the xylem pole pericycle (XPP) that are triggered by the expression of auxin-responsive transcription factors (De Rybel et al., 2010; Goh et al., 2012). After specification, the nuclei in the two founder cells undergo F-actin-dependent nuclear migrations toward the shared wall (Vilches Barro et al., 2019). F-actin depolymerization, either with latrunculin B treatment or tissue-specific expression of the F-actin disrupting DeActs construct (Harterink et al., 2017), disrupts nuclear migration, leading to symmetric division and defective development of the lateral root primordia (Figure 1C). How the two nuclear migrations are oriented in opposite directions toward the shared wall remains unknown. The first zygotic division in *Arabidopsis* is another ACD without a known polar regulator. Kimata et al. used two-photon microscopy and pharmacological treatments to show that the nucleus migrates toward the apical pole in an F-actin-dependent manner before this ACD (Kimata et al., 2016) (Figure 1A). The regulators that are responsible for F-actin reorganization prior to nuclear migration remain unknown.

### Future directions in polarity-mediated organelle positioning

We now have a roster of well-documented, developmentally important ACDs that depend on polarized nuclear migrations, but we remain far from a truly mechanistic understanding of this process. We believe that one of the first steps toward this goal should be to identify the cytoskeletal regulators, motor proteins, and polarized linkers that are required for each of these nuclear migrations. From the published work, it is reasonable to conclude that there is no single mechanism controlling nuclear migrations across asymmetrically dividing cells in plants. However, there is an opportunity to leverage our knowledge of these diverse nuclear migrations to identify the common regulators that may be shared across cell types. For example, the myosin myosin XI-I was shown to be essential for the post-division, F-actin-dependent nuclear migration in the *Arabidopsis* stomatal lineage (Muroyama et al., 2020). Myosin XI-I was first identified as a nuclear envelope-localized myosin that was required for F-actin-dependent nuclear shape changes and nuclear movement in non-proliferative cell types (Tamura et al., 2013), hinting at a potential shared function. Although the closest myosin XI-I homolog in maize, OPAQUE1, is not required for nuclear migration in maize SMCs (Nan et al., 2023b), could myosin XI-I regulate other F-actin-dependent nuclear migrations in *Arabidopsis*?

In the same vein, several kinesins, including members of the kinesin 14 family (KCBP and KCH) and the Armadillo Repeat-Containing Kinesins (ARKs), are required for nuclear transport in *P. patens* and rice and are, therefore, excellent potential candidates for kinesins that could drive pre-division nuclear migration in the *Arabidopsis* stomatal lineage (Frey et al., 2010; Miki et al., 2015; Yamada et al., 2017; Yamada and Goshima, 2018). In fact, KIN14H and KIN14G, members of the kinesin 14 family in *Arabidopsis*, were recently shown to control microtubule-dependent nuclear migration in growing pollen tubes (Wang et al., 2024). The same approach could be taken to define the proteins that link the cytoskeleton to the nuclear envelope. The obvious candidates for these are WIT, WIP, and SUN, components of the LINC complex that bridges the F-actin cytoskeleton and nuclear envelope. Indeed, myosin XI-I localization to the nuclear envelope depends on WIT1/WIT2, and loss-of-function alleles in the LINC components negatively impact nuclear movement in non-proliferative cell types (Tamura et al., 2013; Zhou et al., 2014; Zhou and Meier, 2014; Moser et al., 2020; Brueggeman et al., 2022).

Beyond identifying additional regulators, there are numerous broader questions that warrant additional investigation. How do polar domains locally control cytoskeletal organization? How is cytoskeleton reorganization in polarized cells coupled to the cell cycle? How is cell morphology coupled with the geometry of the cytoskeleton array to control the directionality of nuclear movement? We are particularly intrigued by the prospect that self-organization may allow asymmetrically dividing cells to robustly reorient their cytoskeletons in response to an initial and highly localized symmetry-breaking event. Programs that model how a cytoskeletal array evolves over time in a cell of defined geometry will be particularly useful for these efforts (Saltini and Deinum, 2024).

**FIGURE 2:**
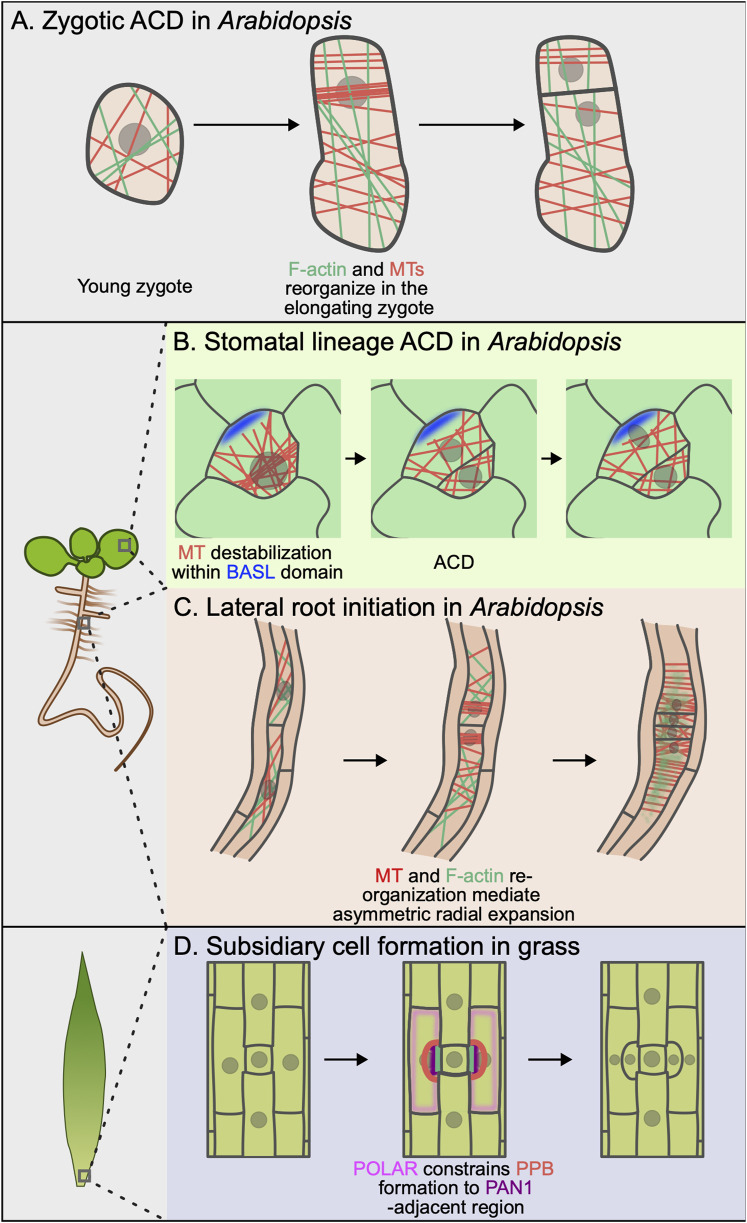
Cytoskeletal rearrangement and cell polarity during asymmetric cell division. **(A)** Upon fertilization, F-actin (green) and microtubules (red) reorganize in the elongating zygote. This reorganization establishes a polarization axis and is important for F-actin–mediated asymmetric organelle localization. **(B)** Cortical BASL (blue) locally destabilizes microtubules (red) within the polar domain, preventing preprophase band (PPB) formation at this site. This ensures asymmetric inheritance of the polar domain by the stomatal lineage ground cell (SLGC) after ACD. **(C)** During lateral root initiation, both F-actin (green) and microtubules (red) undergo dynamic rearrangement that control asymmetric radial expansion in founder cells. **(D)** The localization of PAN1 (purple) excludes cortical POLAR localization (pink). In turn, POLAR blocks TAN1 localization, ensuring proper PPB (red) placement.

Finally, whether polarity controls the active partitioning of all of the other organelles in the cell during asymmetric cell division in plants remains mostly unexplored. Recent work from the Ueda group has made initial progress by characterizing vacuole and mitochondria positioning during the initial ACD in the *Arabidopsis* zygote. Upon fertilization, the large vacuole shrinks and redistributes across the apical and basal regions (Kimata et al., 2019). As the zygote elongates, the vacuole forms a thin tubular structure around the migrating nucleus and only enlarges at the basal end, resulting in an asymmetric vacuole distribution in the fully elongated zygote. Vacuole reorganization depends on longitudinal F-actin arrays (Figure 2A) and, intriguingly, proper vacuole distribution is necessary for nuclear positioning. In the *shoot gravitropism* (*sgr2-1*) mutant, the vacuole is misshapen and less mobile, altering nuclear migration and division orientation (Kimata et al., 2019). By revealing a surprising interplay between the positioning of different organelles, this work serves as a strong motivator to further explore these relationships in other cell types.

The same group examined the dynamics of mitochondria during this zygotic division and found that polarized F-actin distribution concentrates mitochondria in the apical cell (Kimata et al., 2020). Molecular regulators of this process remain unknown, although myosin XI family members, such as myosin XI-K, myosin XI-1, and myosin XI-B, have been shown to control mitochondria motility in non-proliferative cell types and would be excellent candidates for future studies (Avisar et al., 2008; Peremyslov et al., 2008; Prokhnevsky et al., 2008). The functional significance of regulated mitochondria inheritance in plants remains unknown, but it is worth noting that there is a growing body of work from animals and fungi that implicate asymmetric mitochondria partitioning in proliferative potential, cell fate decisions, and general cell function (Katajisto et al., 2015; Döhla et al., 2022; Loeffler et al., 2022; Sun et al., 2023). It will be fascinating to determine whether asymmetric segregation of mitochondria and other organelles have similar functional consequences following ACD in plants.

## Polarized changes to cytoskeletal organization promote morphological changes

Cell morphology is, itself, a major determinant of division orientation in plants. This is primarily due to the fact that, in the absence of other inputs, many plant cells will tend to divide along the axis of maximal tensile stress (Louveaux et al., 2016). Therefore, division orientation in some tissues can be accurately predicted by knowing both the cell shape and pre-division nuclear position (Louveaux et al., 2016; Höfler et al., 2024). As such, polarity-mediated changes to cell morphology and expansion could be reasonably predicted to be important regulators of ACD orientation.

### Polarized cell expansion before ACD

There is strong evidence that morphological changes are required for proper division orientation for two of the ACDs we have discussed: the first division of the one-cell *Arabidopsis* zygote and the paired ACDs in lateral root founder cells (Figure 2). Pre-division anisotropic growth in both of these cells requires dynamic rearrangements of both F-actin and microtubules, the molecular underpinnings of which we are just beginning to understand. In the one-cell zygote, microtubules form a subapical transverse ring and spiral cortical array at the zygote base, while F-actin accumulates at the apical tip and aligns longitudinally along the apicobasal axis (Kimata et al., 2016; Hiromoto et al., 2023). Microtubules are required for the dramatic cell elongation that precedes ACD but not for ensuring that the apical daughter is smaller (Figure 2A). Latrunculin B treatment also impaired cell elongation, although it remains unclear whether this points to a direct role for F-actin in the elongation or whether the failure to elongate is a secondary consequence of failed nuclear migration.

Lateral root founder cells undergo an asymmetric radial expansion that accompanies pre-division nuclear migration (Vilches Barro et al., 2019). As the founder cells expand, cortical microtubules reorganize in two spatially defined domains. Microtubules are relatively isotropic in the “central” region, which is the side of the cell facing the shared wall between founder cells. In contrast, microtubules are organized in transverse arrays in the peripheral domain at the opposite end of the cell. Cell expansion is restricted in this peripheral domain, leading to the observed asymmetric expansion (Figure 2C). Pharmacological or genetic disruption of the microtubule arrays in these cells leads to isotropic cell expansion, indicating that the transverse cortical microtubules locally constrain expansion. Interestingly, F-actin depolymerization also disrupts asymmetric radial expansion, indicating that there is coupling between F-actin and microtubule organization. In sum, these examples reinforce that coordination between polarized cell expansion and nuclear migration can be critical for ACD orientation. These data also point to a still mysterious interplay between the F-actin and microtubule cytoskeletons. Identification of the direct cytoskeletal regulators responsible for the formation of these polarized arrays will begin to shed light on this important question.

### Is local control of cell expansion a shared function across polar domains?

Polar domains are associated with local cell expansion and cell wall remodeling in several developmental contexts, such as pavement cell lobing, trichome morphogenesis, and root hair initiation (Fu et al., 2005; Yanagisawa et al., 2018; Denninger et al., 2019; Lauster et al., 2022). In all of these cases, polarized ROP domains locally alter F-actin and microtubule organization to drive morphogenesis. Taken together with the previously discussed examples of asymmetric cell expansion in the *Arabidopsis* zygote and lateral root founder cells, it is tempting to speculate that the polar domains that control stomatal development might also locally control cell expansion. However, although the PAN1 site in SMCs polarize ROPs and there is some minimal bulging at this site (Panteris et al., 2018), there is no phenotypic evidence indicating that polarized cell growth at the SMC/GMC contact site precedes ACD in the maize leaf.

Intriguingly, BASL preferentially localizes to lobed regions of the membrane when ectopically expressed in tobacco BY-2 cells and *Arabidopsis* (Mansfield et al., 2018; Chan et al., 2020). The position of the polarized BASL domain is also correlated with the overall growth axis when expressed in BY-2 cells, and BASL overexpression in the hypocotyl epidermis induces ectopic lobing (Dong et al., 2009). While these data are consistent with a model where BASL promotes local cell expansion, it remains unclear whether BASL normally has this activity in the stomatal lineage and what the functional significance would be. Time-lapse data of BASL polarization in the stomatal lineage has not shown that BASL polarization increases expansion before cell division, but careful, quantitative analyses will be required to definitively evaluate this hypothesis. A deeper understanding of the cytoskeleton-associated proteins that may co-localize with division-associated polar sites would also open new avenues into this question.

## Polarity-mediated control of division plane placement

Thus far, we have discussed ways that polarized cells can regulate ACD by controlling the cytoskeleton during interphase. While cytoskeleton-dependent effects on nuclear position and cell morphology inform division orientation, the position of the new cell wall is ultimately determined by the phragmoplast, an F-actin and microtubule-based structure that guides membrane deposition to separate the two daughter cells during cytokinesis. In angiosperms, phragmoplast expansion follows the position of a transient band of cortical microtubules called the preprophase band (PPB), which forms around the nucleus in late G2. Even though the PPB itself is disassembled upon entry into mitosis, it recruits a suite of proteins, such as TANGLED, POK1/2, and AIR9, that remain at the plasma membrane in a band called the cortical division zone (CDZ) (Rasmussen and Bellinger, 2018). It is these CDZ proteins that help guide the phragmoplast during cytokinesis to control formation of the new cell wall.

There are numerous examples of polar domains controlling ACD orientation in bacteria, fungi and animals (Thanbichler and Shapiro, 2006; Kiekebusch et al., 2012; Venkei and Yamashita, 2018; Miller et al., 2020). Specific mechanisms vary by species, but they all have a means of regulating mitotic cytoskeletal organization via polar domains. Inspired by this large body of literature and the fact that many polarity mutants exhibit division orientation defects, it has been speculated that similar mechanisms might exist in plants. Excitingly, several recent studies have found evidence that such pathways exist in plants, providing an experimental foothold into this long-standing question.

### Polar domains specify division plane placement during stomatal development

The strongest evidence for polarity-mediated control of cell division machinery comes from studies of stomatal formation (Figure 2). A recent study in *B. distachyon* SMCs found that opposing polar domains specify the localization of the phragmoplast-guiding TANGLED1 (BdTAN1) at the plasma membrane (Zhang et al., 2022). The PAN1 homolog, BdPAN1, is recruited to the same SMC/GMC interface in *B. distachyon* as PAN1 in maize. Most of the remaining plasma membrane forms an opposing polar domain that recruits BdPOLAR. Zhang et al. found that BdTAN1 is localized to a BdPOLAR-depleted region flanking the BdPAN1 site. BdTAN1 is ectopically recruited to inappropriate sites in *bdpolar* even though nuclear migration proceeds normally in this mutant background. BdTAN1 mistargeting leads to SMC division orientation defects, indicating that 1) nuclear migration is not sufficient to orient divisions in these cells and 2) the BdPOLAR domain can somehow restrict recruitment of an important CDS/Z protein (Figure 2D). How BdTAN1 membrane localization inhibits BdTAN1 recruitment remains to be determined.

In complementary work, polarized BASL was recently shown to locally deplete cortical microtubules to control PPB positioning during stomatal lineage ACDs in *Arabidopsis* (Muroyama et al., 2023) (Figure 2B). The first indication that polarized BASL could constrain division orientation came from time-lapse analysis of ACDs in morphologically varied stomatal progenitors, which showed that a significant fraction of ACDs did not divide along the predicted shortest distance. The authors used cell type-specific microtubule reporters and quantitative analyses of microtubule behavior to show that the plus ends of cortical microtubules rapidly undergo catastrophe within the BASL polar domain. They proposed that this destabilizing effect on microtubules prevents PPB formation within the polar site, thereby ensuring that the division plane avoids cortical BASL. In support of this model, the authors showed in the same study that BASL loses control of division site placement in the *trm678* mutant, which cannot make normal PPBs (Schaefer et al., 2017). In *trm678*, the division plane frequently bisects the polar domain, leading to cortical BASL inheritance in both daughters, associated fate stalling and stomatal patterning defects. Future studies are required to determine if BASL interacts with microtubules directly or if it recruits an unknown effector to impact microtubule dynamics.

### Potential polar regulators of division orientation in the root meristem

Whether other polar domains found outside the stomatal lineage control division orientation through similar interactions with microtubules or microtubule-associated proteins remains to be determined. In the root meristem, the LRR-RLK INFLORESCENCE AND ROOT APICES RECEPTOR KINASE (IRK) is polarly localized along global tissue axes (Campos et al., 2020). SOSEKI family proteins, ancient polar proteins found in the genomes of plants down to bryophytes, similarly show global polarity along tissue axes in the root. IRK and SOSEKI loss-of-function or overexpression mutants show aberrant division patterns, demonstrating a link to cell proliferation (Yoshida et al., 2019; Campos et al., 2020; Rodriguez-Furlan et al., 2022). The mechanisms underlying these developmental phenotypes await further investigation, as does whether these polar domains control microtubule or MAP localization to the membrane. Intriguingly, IP-MS data identified ANGUSTIFOLIA, a protein that influences the organization of cortical microtubule arrays (Kim et al., 2002), as a SOSEKI interactor, although the functional relevance of the interaction remains to be determined (van Dop et al., 2020).

## Conclusions and future directions

Here, we focused on three ways that polar domains can control division orientation via the cytoskeleton: organelle positioning, cell growth, and division plane placement. While we described these topics separately for improved clarity, it is important to keep in mind that there is likely significant cross-talk between these pathways. For example, F-actin disruption blocked nuclear migration in lateral root founder cells but also altered asymmetric cell expansion (Vilches Barro et al., 2019). In some ways, the fact that polarity-mediated changes to cytoskeletal organization impact multiple pathways is unsurprising given our extensive knowledge about the many functions for the cytoskeleton in plant cells. A more compelling approach would be to identify whether there are any regulators that allow polar domains to specifically control a single pathway at a time. Additionally, developing a truly mechanistic understanding will require the development of new tools to target cytoskeletal arrays with subcellular resolution, as has been achieved using optogenetics in mammalian cells (Wittmann et al., 2020). Future work that adapts these or other technologies for precise manipulation of subcellular cytoskeletal dynamics in plants will be a huge boon to the field.

Because most of these division-associated polar proteins are predicted to function primarily as scaffolds, a better catalogue of recruited and activated effectors would help address this question. A recent study that used proximity labeling to identify polarity-associated proteins in the *Arabidopsis* stomatal lineage identified some interesting candidates linking OPL2 to the microtubule cytoskeleton, highlighting how new approaches can be used to tackle these problems (Wallner et al., 2024).

In addition to the pathways covered here, there are a number of exciting future directions that remain mostly unexplored. To conclude, we pose some of the open questions that we are most excited by.

1) How is directionality of nuclear movement robustly controlled during ACD?
2) Does the organization of the microtubule and/or F-actin array influence where the polar domain will form?
3) How are dynamic rearrangements of polarized cytoskeletal arrays controlled by progression through the cell cycle?
4) What is the role for cell mechanics in polarization in different cell types? Do mechanical feedback loops ensure robust polarization and ACD orientation?

Deepening our understanding of the relationship between polarity and the cytoskeleton across a variety of developmental contexts will continue to shed light on which components represent common polarity modules and which have evolved for specific morphogenetic functions.
